# Tissue Inhibitor of Metalloproteinase-3 Knockout Mice Exhibit Enhanced Energy Expenditure through Thermogenesis

**DOI:** 10.1371/journal.pone.0094930

**Published:** 2014-04-15

**Authors:** Yohsuke Hanaoka, Osamu Yasuda, Hirofumi Soejima, Keishi Miyata, Eiichiro Yamamoto, Yasuhiro Izumiya, Nobuyo Maeda, Mitsuru Ohishi, Hiromi Rakugi, Yuichi Oike, Shokei Kim-Mitsuyama, Hisao Ogawa

**Affiliations:** 1 Department of Cardiovascular Medicine, Graduate School of Medical Sciences, Kumamoto University, Kumamoto, Japan; 2 Department of Cardiovascular Clinical and Translational Research, Kumamoto University Hospital, Kumamoto University, Kumamoto, Japan; 3 Department of Molecular Genetics, Graduate School of Medical Sciences, Kumamoto University, Kumamoto, Japan; 4 Department of Pathology and Laboratory Medicine, School of Medicine, The University of North Carolina at Chapel Hill, Chapel Hill, North Carolina, United States of America; 5 Department of Cardiovascular Medicine and Hypertension, Graduate School of Medical and Dental Science, Kagoshima University, Kagoshima, Japan; 6 Department of Geriatric Medicine and Nephrology, Osaka University Graduate School of Medicine, Suita, Japan; 7 Department of Pharmacology and Molecular Therapeutics, Graduate School of Medical Science, Kumamoto University, Kumamoto, Japan; University of Santiago de Compostela School of Medicine - CIMUS, Spain

## Abstract

Tissue inhibitors of metalloproteinases (TIMPs) regulate matrix metalloproteinase activity and maintain extracellular matrix homeostasis. Although TIMP-3 has multiple functions (e.g., apoptosis, inhibition of VEGF binding to VEGF receptor, and inhibition of TNFα converting enzyme), its roles in thermogenesis and metabolism, which influence energy expenditure and can lead to the development of metabolic disorders when dysregulated, are poorly understood. This study aimed to determine whether TIMP-3 is implicated in metabolism by analyzing TIMP-3 knockout (KO) mice. TIMP-3 KO mice had higher body temperature, oxygen consumption, and carbon dioxide production than wild-type (WT) mice, although there were no differences in food intake and locomotor activity. These results suggest that metabolism is enhanced in TIMP-3 KO mice. Real-time PCR analysis showed that the expression of PPAR-δ, UCP-2, NRF-1 and NRF-2 in soleus muscle, and PGC-1α and UCP-2 in gastrocnemius muscle, was higher in TIMP-3 KO mice than in WT mice, suggesting that TIMP-3 deficiency may increase mitochondrial activity. When exposed to cold for 8 hours to induce thermogenesis, TIMP-3 KO mice had a higher body temperature than WT mice. In the treadmill test, oxygen consumption and carbon dioxide production were higher in TIMP-3 KO mice both before and after starting exercise, and the difference was more pronounced after starting exercise. Our findings suggest that TIMP-3 KO mice exhibit enhanced metabolism, as reflected by a higher body temperature than WT mice, possibly due to increased mitochondrial activity. Given that TIMP-3 deficiency increases energy expenditure, TIMP-3 may present a novel therapeutic target for preventing metabolic disorders.

## Introduction

Metabolic syndrome, a chronic disorder with increasing incidence and prevalence worldwide, is associated with an increased risk of developing atherosclerotic disease and presents a major public health/medical economics concern. Accordingly, the prevention of metabolic syndrome is an urgent issue.

Mitochondrial function is compromised in metabolic syndrome, which is why it is often referred to as a mitochondrial disease [1]. Mitochondria are important for maintaining the homeostasis of skeletal muscle fibers and producing energy in response to demand via glycolysis and β-oxidation. These processes produce NADH, which is used to generate the electrochemical membrane potential through the electron transport chain. In a reaction known as oxidative phosphorylation, the membrane potential of mitochondria drives the phosphorylation of ADP to ATP. Compromised mitochondrial function is associated with various diseases, as well as aging [2].

The tissue inhibitor of metalloproteinase (TIMP) family consists of four members, TIMP-1 to -4. TIMPs regulate matrix metalloproteinase (MMP) activity and maintain extracellular matrix homeostasis. TIMP-3 is unique among TIMPs in that it is bound to the extracellular matrix. TIMP-3 has multiple functions, including inhibiting MMPs [3], which have a broad range of substrates such as members of the ADAM (a disintegrin and metalloprotease) and ADAMTS (ADAM with thrombospondin motifs) family [4], [5], [6]; inhibiting VEGF-mediated angiogenesis and neovascularization by blocking the binding of VEGF to VEGFR-2 [7] and inhibiting tumor necrosis factor-α converting enzyme (TACE, ADAM-17) by binding to its N-terminal domain and blocking the release of TNF-α [8], which promotes apoptosis by binding to the TNF death receptor [9].

Extracellular matrix remodeling impacts adipocyte differentiation [10]. MMP expression is modulated in adipose tissue of mice with diet-induced obesity, as well as genetically obese mice [11], [12], [13]. The expression of TIMPs is also regulated during adipose tissue development [11], [13]. Of the four TIMPs, TIMP-3 is implicated in adipocyte differentiation [14]. Moreover, one study reported that TIMP-1 deficient mice are protected from obesity [15]. However, the roles of TIMP-3 in thermogenesis and metabolism, which influence energy expenditure and can lead to the development of metabolic disorders when dysregulated, are poorly understood. This study aimed to examine the role of TIMP-3 in metabolism by analyzing TIMP-3 knockout (KO) mice.

## Methods

### Animals

All experimental protocols were approved by the Kumamoto University Ethics Review Committee for Animal Experimentation. TIMP-3 KO mice were generated by gene targeting as described previously [16]. Mouse genotypes were determined by PCR using tail DNA [16]. Male TIMP-3 KO mice and wild-type (WT) mice with a C57BL/6J background were used for all experiments. All mice were bred in housing with automatically controlled lighting (light, 7∶00–19∶00; dark, 19∶00–7∶00), and a stably maintained temperature of 22±1–2°°C. Mice were fed a normal chow diet (CE-2, CLEA, Tokyo, Japan).

### Metabolic Measurements

Oxygen consumption (VO_2_), carbon dioxide production (VCO_2_), the respiratory exchange ratio (RER) and activity levels were determined (Light time; 7∶00–19∶00, Dark time; 19∶00–7∶00, air flow rate 0.50 L/min) using an O_2_/CO_2_ metabolic measuring system (Model MK-5000, Muromachi Kikai, Tokyo, Japan). Body temperature was measured using a rectal probe attached to a digital thermometer (Thermalert TH-5, Physitemp, Clifton, New Jersey, USA). For cold exposure experiments, mice were placed in individual cages under fasting conditions for 8 h at 4°C.

### Quantitative Real-time PCR

Total RNA was isolated from 8-month-old mice using a RNeasy fibrous minikit and RNeasy fibrous tissue mini kit (Qiagen, Hilden, Germany), according to the manufacturer’s instructions. Complementary DNA was generated using the ThermoScript RT-PCR System (Invitrogen, Carlsbad, CA, USA). Quantitative real-time PCR was performed with the iCycler iQ Real-Time PCR Detection System (BIO-RAD) using SYBR Green I as a double-stranded DNA-specific dye, according to the manufacturer’s instructions (Applied Biosystems, Foster City, CA, USA).

### Western Blot Analysis

To determine the expression of mitochondria-related genes in TIMP-3 KO mice, soleus muscle was harvested and homogenized. Ten µg of protein from each sample was denatured with 4X Sample Buffer Solution with 3-mercapto-1,2-propanediol (Wako, Osaka, Japan) and subjected to SDS-PAGE. Separated proteins were transferred to a polyvinylidene difluoride (PVDF) membrane followed by blocking with PVDF Blocking Reagent for Can Get Signal (Toyobo, Osaka, Japan) for 1 h at room temperature. After blocking, membranes were probed with primary antibodies for 1 h at room temperature, secondary antibody for 1 h at room temperature, and visualized with the ECL detection system. The intensity of each band was quantified using NIH ImageJ analysis software v1.61 (National Institutes of Health, Bethesda, MD) and normalized to GAPDH signal. The antibodies used were anti-UCP-2 (1∶1000; Proteintech, Chicago, IL, USA), anti-NRF-1 (1∶1000; abcam, Cambridge, UK), anti-NRF-2 (1∶500; abcam, Cambridge, UK), anti-VDAC (1∶1000; Cell Signaling Technology, Danvers, MA, USA), anti-COX4 (1∶1000; GeneTex, Irvine, CA, USA) and anti-GAPDH (1∶30000; Cell Signaling Technology, Danvers, MA, USA).

### Immunohistochemistry

Gastrocnemius muscle tissue was harvested and frozen in liquid nitrogen. Muscle tissue was then cut into 10 µm sections. After drying at room temperature and washing in PBS 3 times for 5 min, slices were fixed with cold acetone for 20 min, and washed in PBS 3 times for 5 min. Sections were blocked with PBS including 2% BSA for 20 min at room temperature, and incubated with rat anti-mouse CD31 antibody (1∶40, BD Biosciences, San Jose, CA, USA) for 1 h. After incubating with goat anti-rat secondary antibody (Alexa Flour 555, 1∶500; Invitrogen, Carlsbad, CA, USA) for 30 min under shaking and washing with PBS, samples were stained with DAPI using VECTASHIELD (Vector Laboratories, Inc., Burlingame, CA, USA). Fluorescence was visualized with a Biozero fluorescence microscope (Keyence, Osaka, Japan).

### Statistical Analysis

Statistical analysis was performed with the Mann Whitney non-parametrical t-test. P≤0.05 was considered statistically significant. Results are expressed as mean ± S.D.

## Results

**Figure 1:**
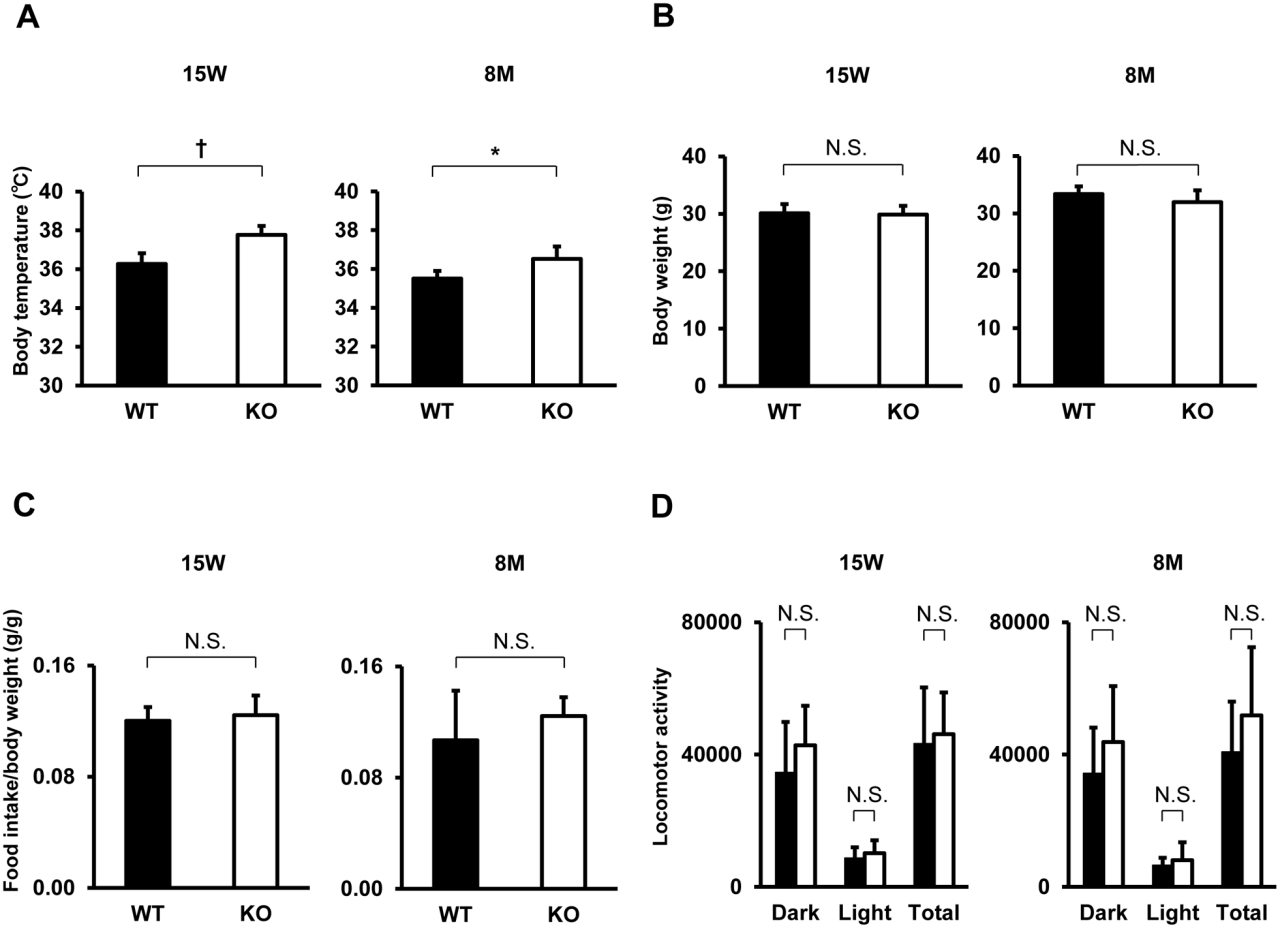
Metabolic parameters in TIMP-3 KO and wild type mice. Body temperature (A), body weight (B), food intake per body weight (C) and locomotor activity (D) in TIMP-3 knockout (KO) and wild type (WT) mice at 15 weeks or 8 months of age are presented as mean ± SD (n = 5–7/group). ^†^p<0.01, ^‡^p<0.001.

**Figure 2:**
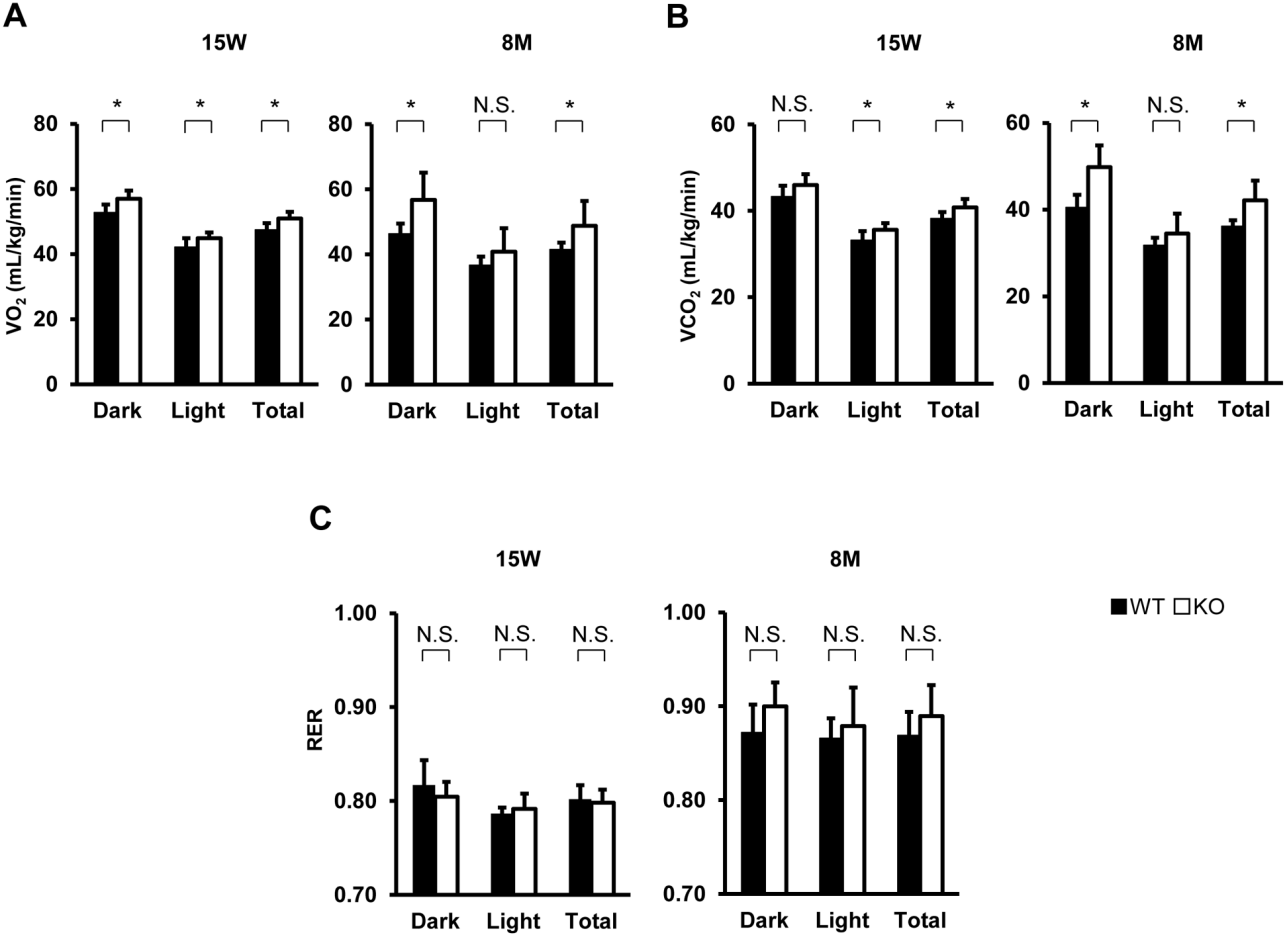
Respiratory parameters in TIMP-3 KO and wild type mice. Oxygen consumption (VO_2_) (A), carbon dioxide production (VCO_2_) (B) and respiratory exchange ratio (RER) (C) in TIMP-3 knockout (KO) and wild type (WT) mice at 15 weeks or 8 months of age are presented as mean ± SD (n = 5–7/group). *p<0.05, ^†^p<0.01.

To investigate the involvement of TIMP-3 in metabolism, we analyzed the basal metabolic rate, locomotor activity, food intake (using a metabolic chamber), body weight and body temperature of WT and TIMP-3 KO mice. TIMP-3 KO mice had a significantly higher body temperature compared to WT mice (Figure 1A). VO_2_ and VCO_2_ were also higher in TIMP-3 KO mice (Figure 2A, 2B), while food intake and locomotor activity did not differ between WT and TIMP-3 KO mice (Figure 1C, 1D). No significant differences were observed in body weight and RER (Figure 1B, 2C). Body surface area estimated by Meeh’s formula did not differ between TIMP-3 KO and WT mice (WT mice vs TIMP-3 KO mice, 95.43±5.68 cm^2^ vs 93.18±6.41 cm^2^; n = 7, N.S.), and body length was also similar (WT mice vs TIMP-3 KO mice, 8.54±0.42 cm vs 8.22±0.36 cm; n = 7, N.S.). These results suggest that metabolism is enhanced in TIMP-3 KO mice.

**Figure 3:**
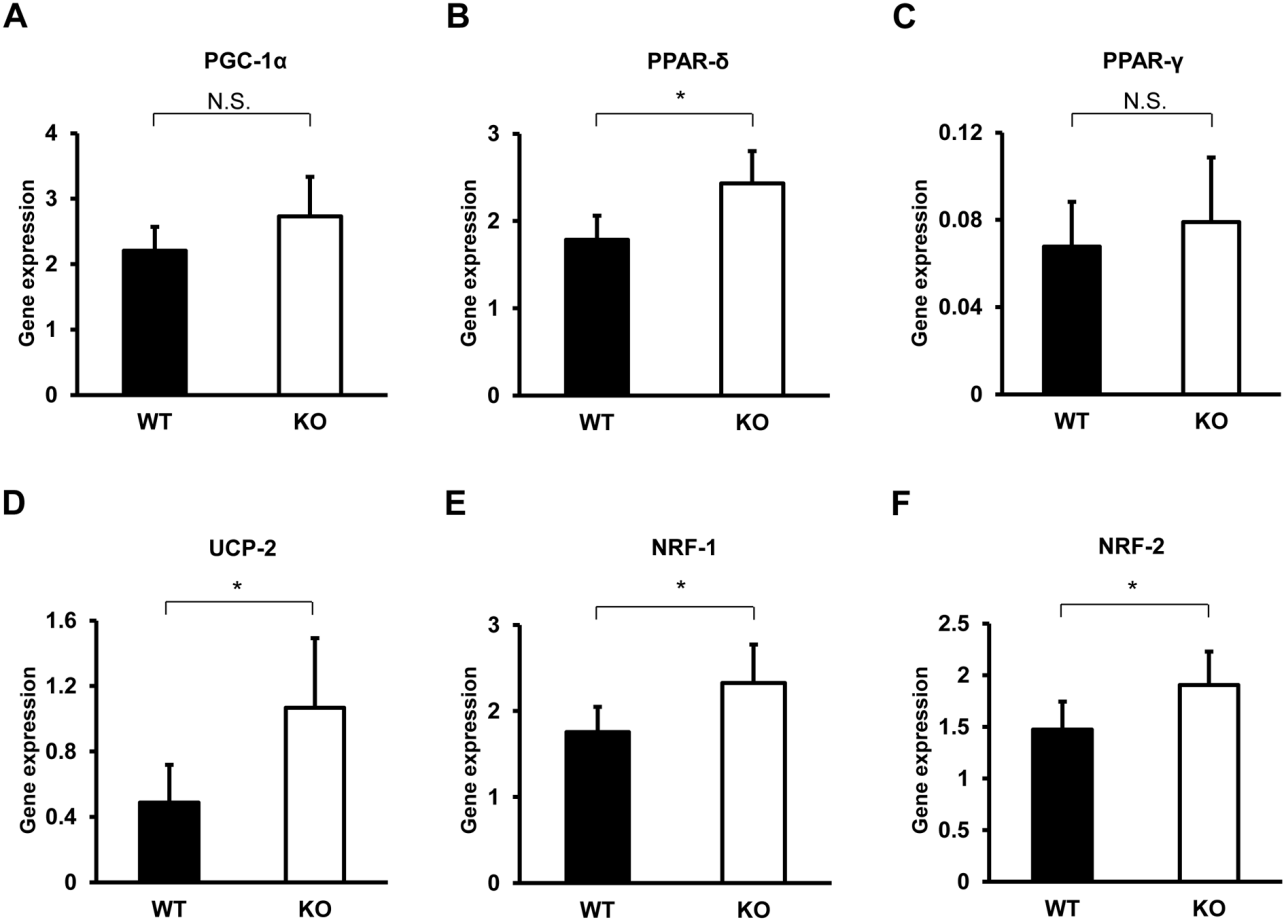
Real-time PCR-based analysis of mitochondrial activity in soleus muscle of TIMP-3 KO and wild type mice. Expression of PGC-1α (A), PPAR-δ (B), PPAR-γ (C), UCP-2 (D), NRF-1 (E) and NRF-2 (F) in TIMP-3 knockout (KO) and wild type (WT) mice is presented as mean ± SD (n = 6–7/group). *p<0.05 ^†^p<0.01.

**Figure 4:**
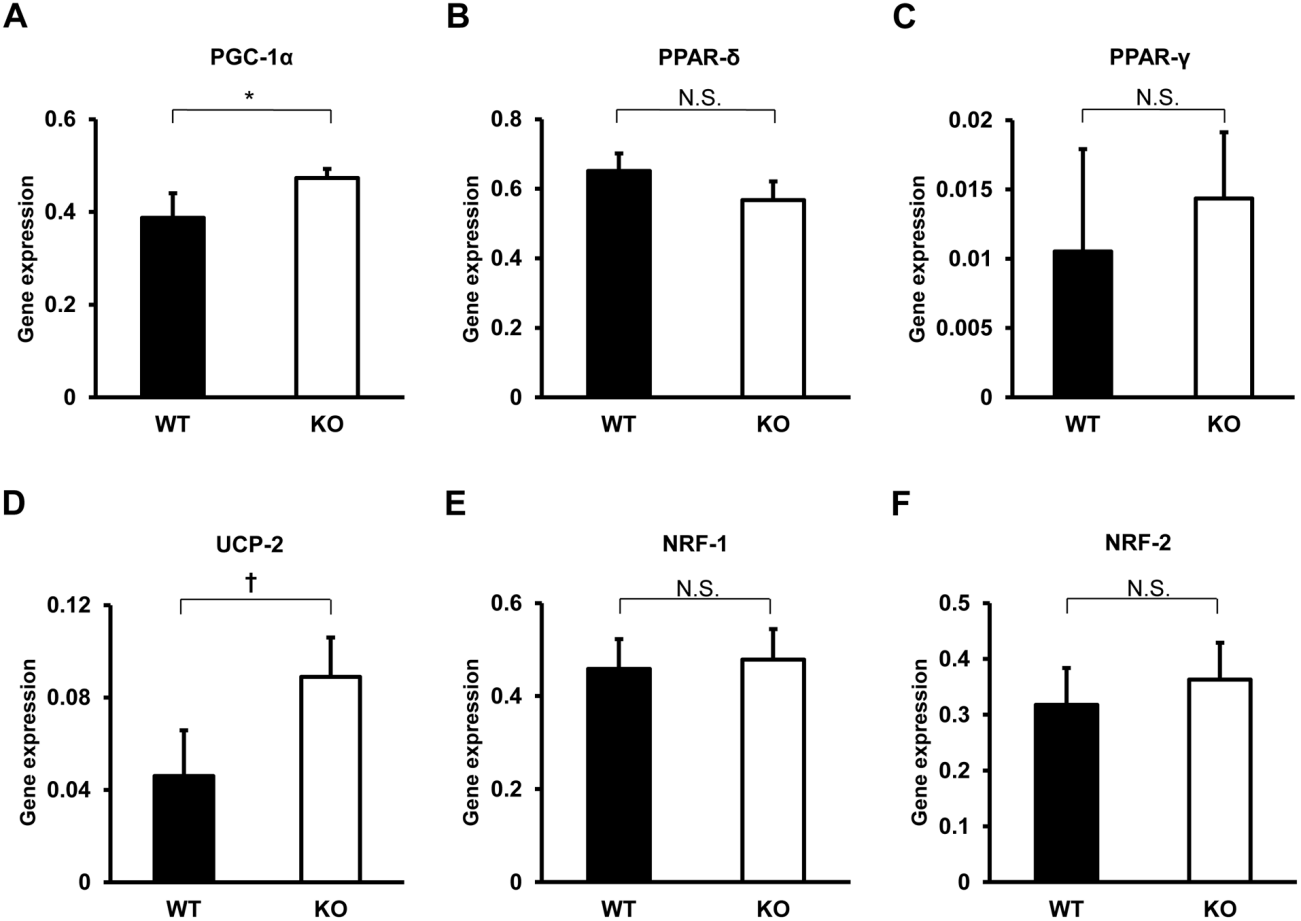
Real-time PCR-based analysis of mitochondrial activity in gastrocnemius muscle of TIMP-3 KO and wild type mice. Expression of PGC-1α (A), PPAR-δ (B), PPAR-γ (C), UCP-2 (D), NRF-1 (E) and NRF-2 (F) in TIMP-3 knockout (KO) and wild type (WT) mice is presented as mean ± SD (n = 6–7/group). ^†^p<0.01.

**Figure 5:**
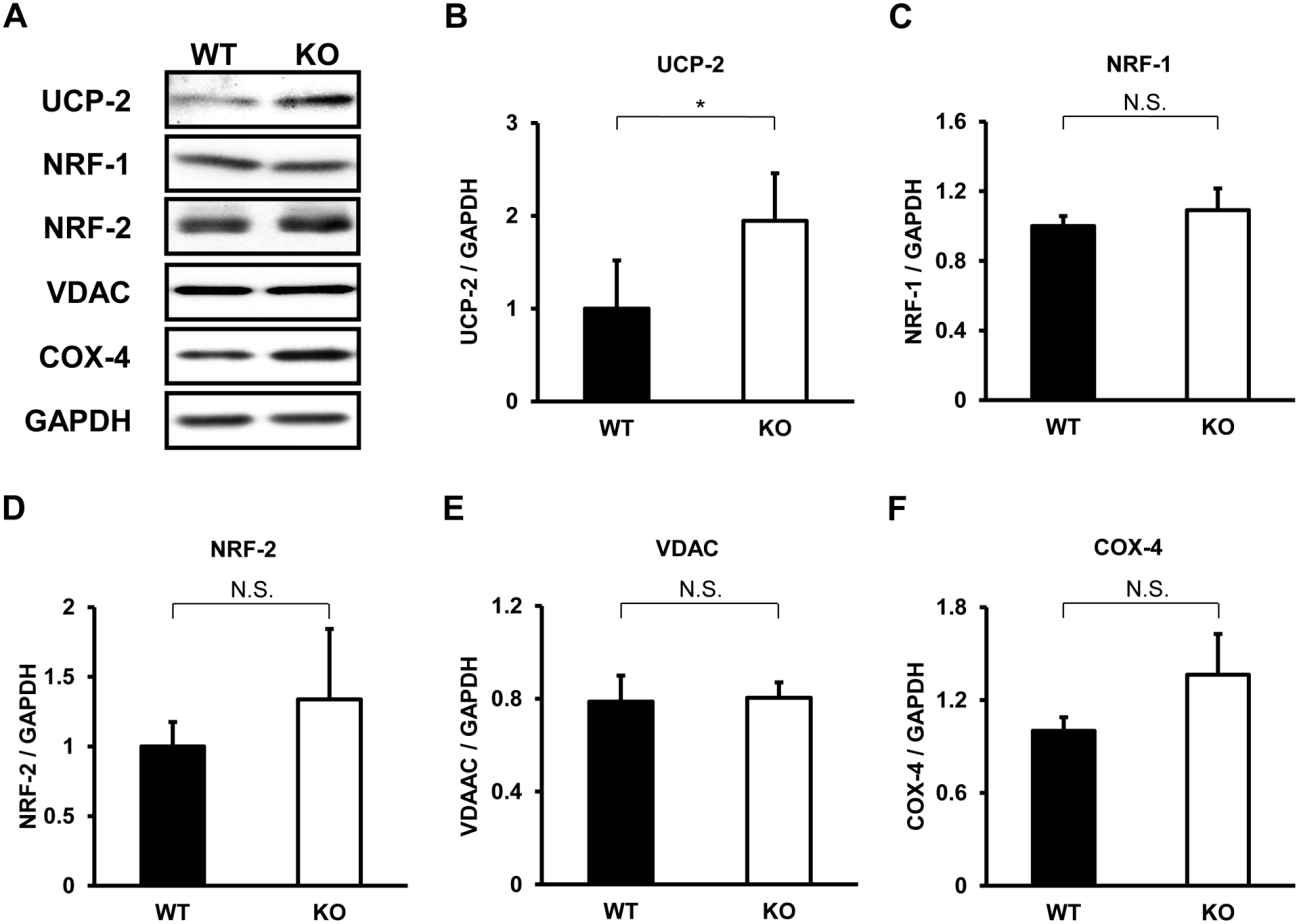
Western blot analysis of mitochondrial proteins in soleus muscle of TIMP-3 KO and wild type mice. Representative Western blot for each mitochondria-related gene (A), and average protein content of UCP-2 (B), NRF-1 (C), NRF-2 (D), VDAC (E) and COX-4 (F). Data are presented as mean ± SD (n = 3/group). *p<0.05.

We next investigated whether the enhanced metabolism in TIMP-3 KO mice has a mitochondrial basis. To this end, quantitative real-time PCR of mitochondria-related genes was performed on RNA isolated from brown adipose tissue, which is involved in energy expenditure under basal conditions, white adipose tissue, soleus muscle and gastrocnemius muscle. In soleus muscle, the expression of PPAR-δ, UCP-2, NRF-1 and NRF-2 was higher in TIMP-3 KO mice relative to WT mice (Figure 3). In gastrocnemius muscle, PGC-1α and UCP-2 expression was higher in TIMP-3 KO mice (Figure 4). The expression of mitochondria-related genes did not significantly differ in brown adipose tissue between WT and TIMP-3 KO mice, nor did it significantly differ between the two strains in white adipose tissue (Figures S1 and S2). Expression of UCP1, which mediates heat generation in brown fat, and Dio2, which activates thyroid hormone, did not differ in brown adipose tissue of WT and TIMP-3 KO mice. In Western blot analysis, UCP-2 expression was significantly higher in soleus muscle of TIMP-3 KO mice compared to WT mice, which was consistent with results from real-time PCR (Figure 5). NRF-1 and NRF-2 expression did not significantly differ between WT and TIMP-3 KO mice. The expression of VDAC and COX IV, marker proteins for mitochondrial content, was similar between WT and TIMP-3 KO mice, indicating that either mitochondrial content is similar, or the difference in content too small to detect, between the two mouse strains.

**Figure 6:**
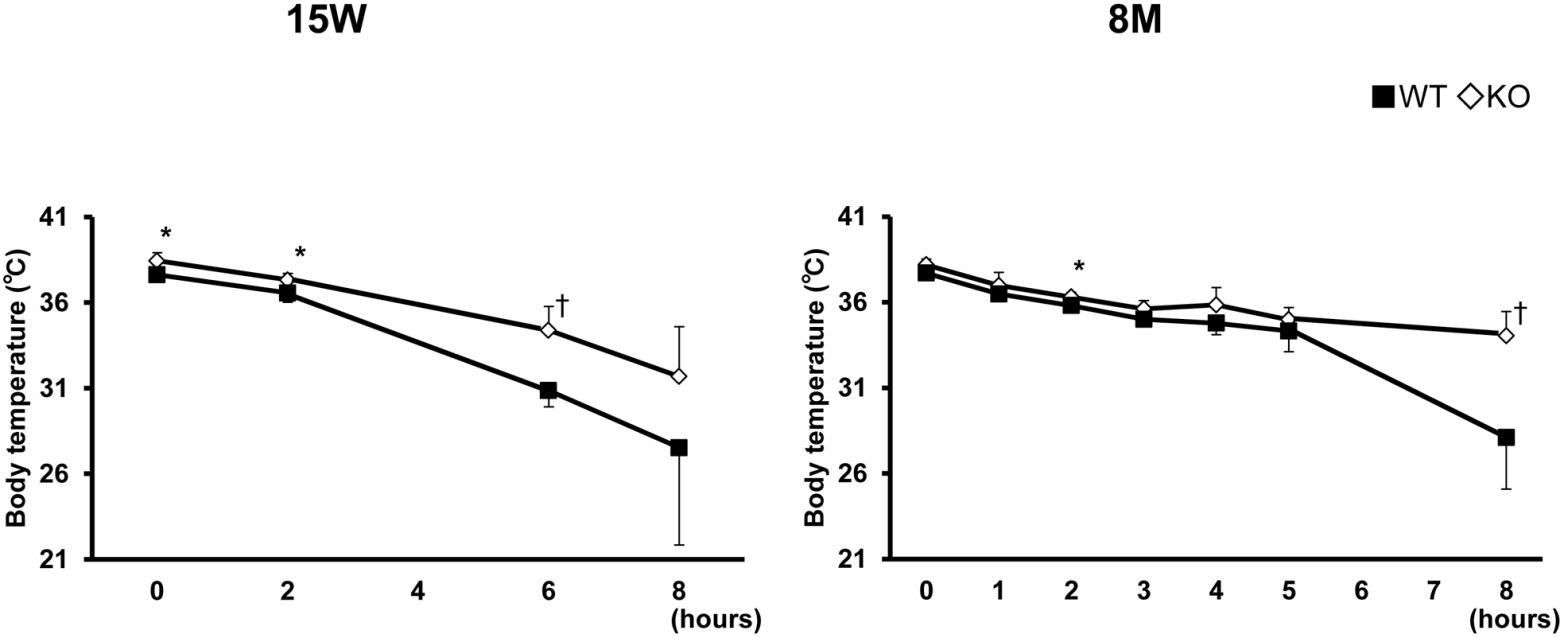
Cold exposure experiments in TIMP-3 KO and wild type mice. Mice at 15 weeks or 8 months of age were exposed to 4°C for 8 hours. Data are presented as mean ± SD (n = 5/group). *p<0.05 ^†^p<0.01.

The expression of endothelium-specific marker genes, Tie-2 and Cadherin (Figure S3), and the number of CD31-positive cells (Figure S4) did not differ between TIMP-3 KO mice and WT mice. These results indicate that the number of blood vessels, which can affect metabolism, was not affected in TIMP-3 KO mice.

Adaptive thermogenesis refers to the production of heat in response to changes in environmental temperature or diet, which protects organisms from cold exposure or dietary changes [17]. To assess whether TIMP-3 plays a role in adaptive thermogenesis, TIMP-3 KO and WT mice were exposed to a cold environment at 4°C for 8 h. Under these conditions, TIMP-3 KO mice had a higher core body temperature than that of WT mice (Figure 6), indicating that TIMP-3 KO mice exhibit enhanced heat production under room temperature and cold environment conditions.

In the metabolic chamber test, TIMP-3 KO mice showed higher VO_2_ and VCO_2_ than WT mice (Figure 2A, 2B). Thus, we next assessed whether VO_2_ and VCO_2_ further increase in response to treadmill-based exercise in TIMP-3 KO mice (Text S1). VO_2_, VCO_2_ and RER were monitored during a 10 m/min treadmill running session, and significant differences were observed in VO_2_ and VCO_2_ in TIMP-3 KO mice before and after starting the exercise (Figure S5A, S5B). As the exercise continued, differences in VO_2_ and VCO_2_ became more pronounced. Based on this finding, we further evaluated body weight after treadmill-based physical training. While body weight decreased after exercise in both TIMP-3 KO and WT mice, relative body weight after exercise tended to decrease more in TIMP-3 KO mice compared to WT mice (Figure S6). Moreover, while running time and distance were noticeably higher in TIMP-3 KO mice compared to WT mice, the difference was not significant (Figure S7).

## Discussion

Numerous studies have investigated the relationship between TIMP members and metabolism. These studies have shown that TIMP-3 inhibits MMP-2 and MMP-9, both of which are involved in adipocyte differentiation [11], [13], [18]; TIMP-3 is a modifier gene for insulin resistance in mice [19] and TIMP-1 KO mice are protected from obesity [15]. However, it is still unclear whether TIMP-3 plays a role in metabolism. Here, we demonstrate for the first time that TIMP-3 deficiency modulates mitochondrial metabolism and body temperature in mice.

Obesity results from an imbalance between energy supply and demand. Energy enters an organism as food, and exits as heat and work. Heat production can be divided into three components: energy expenditure resulting from physical activity; obligatory energy expenditure required for normal functioning of cells and organs and expenditure attributed to adaptive thermogenesis, which is defined as heat production in response to environmental temperature or diet [17]. In this study, we found that expenditure resulting from physical activity in TIMP-3 KO mice is essentially identical to that of WT mice, as evidenced by the lack of a difference in locomotor activity. The obligatory energy expenditure required for basal activity is elevated in TIMP-3 KO mice, as reflected in the significantly higher body temperature compared to WT mice. Expenditure attributed to adaptive thermogenesis was increased in TIMP-3 KO mice compared to WT mice, as evaluated by cold exposure experiments. The higher body temperature of TIMP-3 KO mice in the cold exposure experiments can be attributed to shivering and/or mitochondrial activation, since adaptive thermogenesis consists of shivering thermogenesis by skeletal muscle and non-shivering thermogenesis by brown adipose tissue [20].

Body temperature is known to be related to basal metabolism; in fact, the metabolic rate rises 13% with each 1°C increase in body temperature in humans, suggesting that a high body temperature is indicative of increased basal metabolism. In this study, locomotor activity, food intake, body length and surface area, which affect heat dissipation, did not differ between TIMP-3 KO mice and WT mice. Thus, the body temperature of TIMP-3 KO mice reflects basal metabolism. Accordingly, the higher body temperature in TIMP-3 KO mice compared to WT mice is evidence that TIMP-3 KO mice have elevated basal metabolism. Normally, higher energy expenditure with equal locomotor activity and food intake render animals lean. However, body weight did not significantly differ between WT and TIMP-3 KO mice. This may be due to the difficulty of accurately measuring food intake, or the small sample size of this study.

Oxygen is transported to systemic tissues by the cardiovascular system, and is used for the production of ATP in mitochondria. VO_2_ can be used to determine the amount of energy expenditure, since almost all energy in the body is produced aerobically. Our finding that VO_2_ and VCO_2_ are higher in TIMP-3 KO mice relative to WT mice implies that TIMP-3 may be implicated in the regulation of energy expenditure.

Real-time PCR revealed that the expression of PPAR-δ is approximately 1.4-fold higher in skeletal muscle of TIMP-3 KO mice than in WT mice. Despite the lack of a significant difference in running time and distance, both parameters were noticeably increased in TIMP-3 KO mice relative to WT mice. This finding is consistent with that reported in a study by Wang et al., which found that overexpression of PPAR-δ using a transgene increased physical endurance in mice [21]. The lack of a significant difference in our study might be explained by the fact that transgenic mice express much higher levels of PPAR-δ compared to TIMP-3 KO mice.

Significant differences in VO_2_ and VCO_2_ were observed in the treadmill test in TIMP-3 KO mice both before and after starting the exercise. These differences became more pronounced as time progressed after starting the exercise, suggesting that metabolism in TIMP-3 KO mice increased more than in WT mice with exercise. This may explain the differences in body weight, which tended to be less in TIMP-3 KO mice compared to WT mice after two weeks of the treadmill test.

VEGF exerts metabolic effects, and both its up- and down-regulation control energy metabolism [22], [23], [24]. VEGF has beneficial effects in adipose tissue. For example, it promotes vascularization and thermogenesis to counteract the development of high fat diet-induced obesity [22]. TIMP-3 also inhibits the action of VEGF by blocking its binding to VEGFR-2 [7]. In this study, we examined the number of blood vessels in skeletal muscle of TIMP-3 KO mice and found no difference compared to WT mice. Consistent with this, the expression of endothelial marker genes did not differ between TIMP-3 KO mice and WT mice. The lack of difference in number of vessels in skeletal muscle and adipose tissue, as assessed by immunohistochemical staining vascular endothelial cells, further supports this conclusion. However, these findings do not contradict TIMP-3′s known role in angiogenesis, since differences in angiogenesis in TIMP-3 KO mice were reported in the context of providing an angiogenic stimulus [25]. Our results simply indicate that differences in metabolism between TIMP-3 KO and WT mice are not a reflection of differences in the number of blood vessels. In addition to regulating the number of arteries, TIMP-3 is involved in vascular function via preservation of the extracellular matrix of arteries [26]. Given that vascular function is closely related to thermogenesis [27], it is possible that impaired vascular function contributes to enhanced thermogenesis in TIMP-3 KO mice.

Real-time PCR revealed an increase in the expression of mitochondria-related genes in the skeletal muscle of TIMP-3 KO mice. PGC-1α, a transcriptional co-activator, induces mitochondrial biogenesis, and transgenic mice expressing this gene in muscle tissue exhibit an increased proportion of oxidative muscle fibers and enhanced exercise performance [28]. Muscle-specific PPAR-δ transgenic mice exhibit increased energy metabolism and muscle endurance in the absence of exercise [21], [29], [30]. UCP-2 is widely expressed in mammalian tissues [31], [32], uncouples respiration [33] and plays a role in energy dissipation as heat. Interestingly, the UCP gene resides within a region genetically linked to obesity [31]. NRF-1 is a DNA-binding transcription factor that activates genes involved in mitochondrial biogenesis and function and other fundamental cellular functions. NRF-2 is implicated in the control of basic cellular processes, such as cell cycle progression, protein synthesis and mitochondrial biogenesis [34], [35], [36]. Consistent with the relatively small difference in NRF-1 and NRF-2 expression between WT and TIMP-3 KO mice observed by real-time PCR, no significant difference in their expression was observed by Western blot analysis. Our data suggest that the increased expression of mitochondria-related genes may contribute to the enhanced metabolism observed in TIMP-3 KO mice.

Taken together, our data indicate that TIMP-3 is involved in increasing the metabolic rate and energy expenditure through thermogenesis, and that its deficiency may enhance mitochondrial activity. TIMP-3 may thus present a novel therapeutic target for preventing metabolic disorders.

## Supporting Information

Figure S1 **Real-time PCR-based analysis of mitochondrial activity in brown adipose tissue of TIMP-3 KO and wild type mice.** Expression of PGC-1α (A), PPAR-δ (B), PPAR-γ (C), UCP-1 (D), UCP-2 (E), NRF-1 (F), NRF-2 (G) and Dio-2 (H) in TIMP-3 knockout (KO) and wild type (WT) mice is presented as mean ± SD (n = 6–7/group).
(TIF)

Figure S2 **Real-time PCR-based analysis of mitochondrial activity in white adipose tissue of TIMP-3 KO and wild type mice.** Expression of PGC-1α (A), PPAR-δ (B), PPAR-γ (C), UCP-2 (D), NRF-1 (E) and NRF-2 (F) in TIMP-3 knockout (KO) and wild type (WT) mice is presented as mean ± SD (n = 6–7/group).
(TIF)

Figure S3 **Endothelial gene expression in skeletal muscle of TIMP-3 KO and wild type mice.** Expression of Tie-2 (A) and Cadherin (B) in soleus muscle, and Tie-2 (C) and Cadherin (D) in gastrocnemius muscle, in TIMP-3 knockout (KO) and wild type (WT) mice is presented as mean ± SD (n = 6–7/group).
(TIF)

Figure S4 **Vessel counts in gastrocnemius muscle of TIMP-3 KO and wild type mice.** Immunostaining for CD31, an endothelial cell marker (A), and quantification of vessels based on CD31 expression (B) in soleus muscle of TIMP-3 knockout (KO) and wild type mice.
(TIF)

Figure S5 **Respiratory parameters of TIMP-3 KO and wild type mice during the exercise performance test.** Oxygen consumption (VO_2_) (A) and carbon dioxide production (VCO_2_) (B) in TIMP-3 knockout (KO) and wild type (WT) mice are presented as mean ± SD (n = 7/group). *p<0.05, ^†^p<0.01, ^‡^p<0.001.
(TIF)

Figure S6 **Body weight of TIMP-3 KO and wild type mice before and after exercise.** Net body weight (mean ± SD) (A) and body weight relative to before exercise (mean ± SD) (B) are presented (n = 8/group).
(TIF)

Figure S7 **Physical endurance in TIMP-3 KO and wild type mice.** Running time (A) and distance (B) for TIMP-3 knockout (KO) and wild type (WT) mice are presented as mean ± SD (n = 8/group).
(TIF)

Text S1 **Protocol of exercise performance test and Treadmill-based physical training.**
(DOCX)
