# Supplementary figures and images for: Tissue Inhibitor of Metalloproteinase-3 Knockout Mice Exhibit Enhanced Energy Expenditure through Thermogenesis
Source: PLoS One. 2014 Apr 15;9(4):e94930. doi: 10.1371/journal.pone.0094930 (PMC3988092; doi:10.1371/journal.pone.0094930)

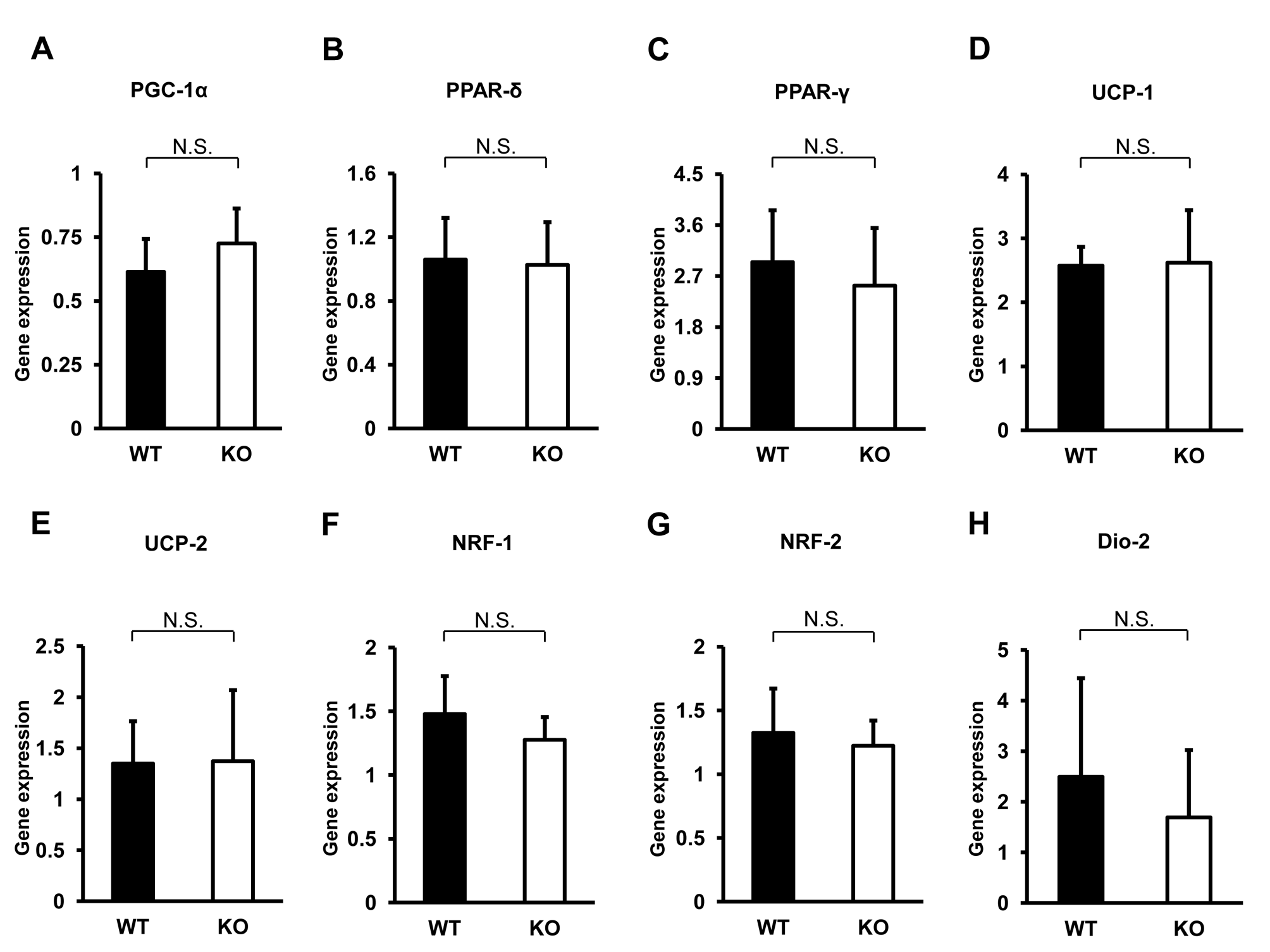

Supplement: Figure S1 — Real-time PCR-based analysis of mitochondrial activity in brown adipose tissue of TIMP-3 KO and wild type mice. Expression of PGC-1α (A), PPAR-δ (B), PPAR-γ (C), UCP-1 (D), UCP-2 (E), NRF-1 (F), NRF-2 (G) and Dio-2 (H) in TIMP-3 knockout (KO) and wild type (WT) mice is presented as mean ± SD (n = 6–7/group). (TIF) [file pone.0094930.s001.tif]

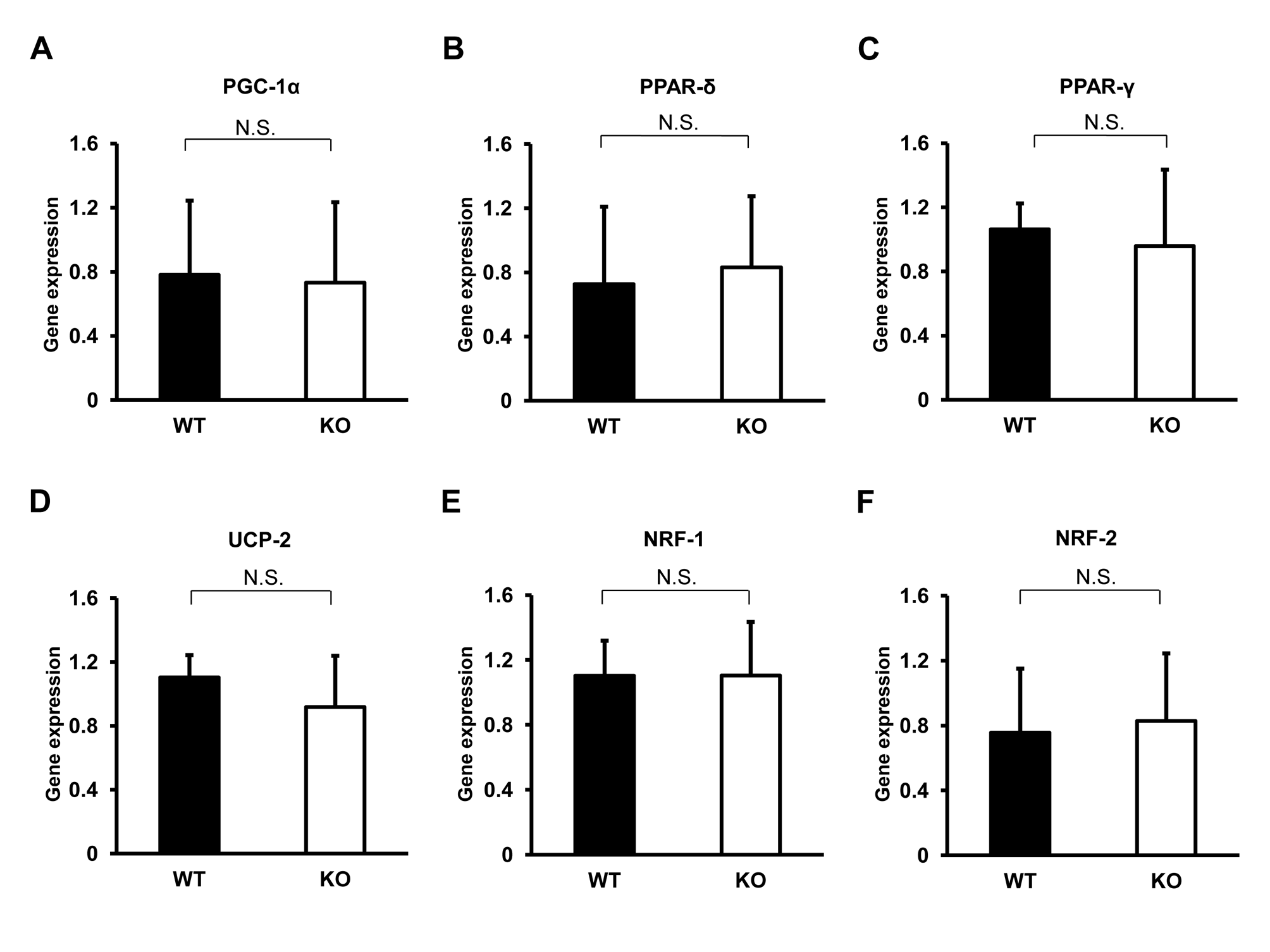

Supplement: Figure S2 — Real-time PCR-based analysis of mitochondrial activity in white adipose tissue of TIMP-3 KO and wild type mice. Expression of PGC-1α (A), PPAR-δ (B), PPAR-γ (C), UCP-2 (D), NRF-1 (E) and NRF-2 (F) in TIMP-3 knockout (KO) and wild type (WT) mice is presented as mean ± SD (n = 6–7/group). (TIF) [file pone.0094930.s002.tif]

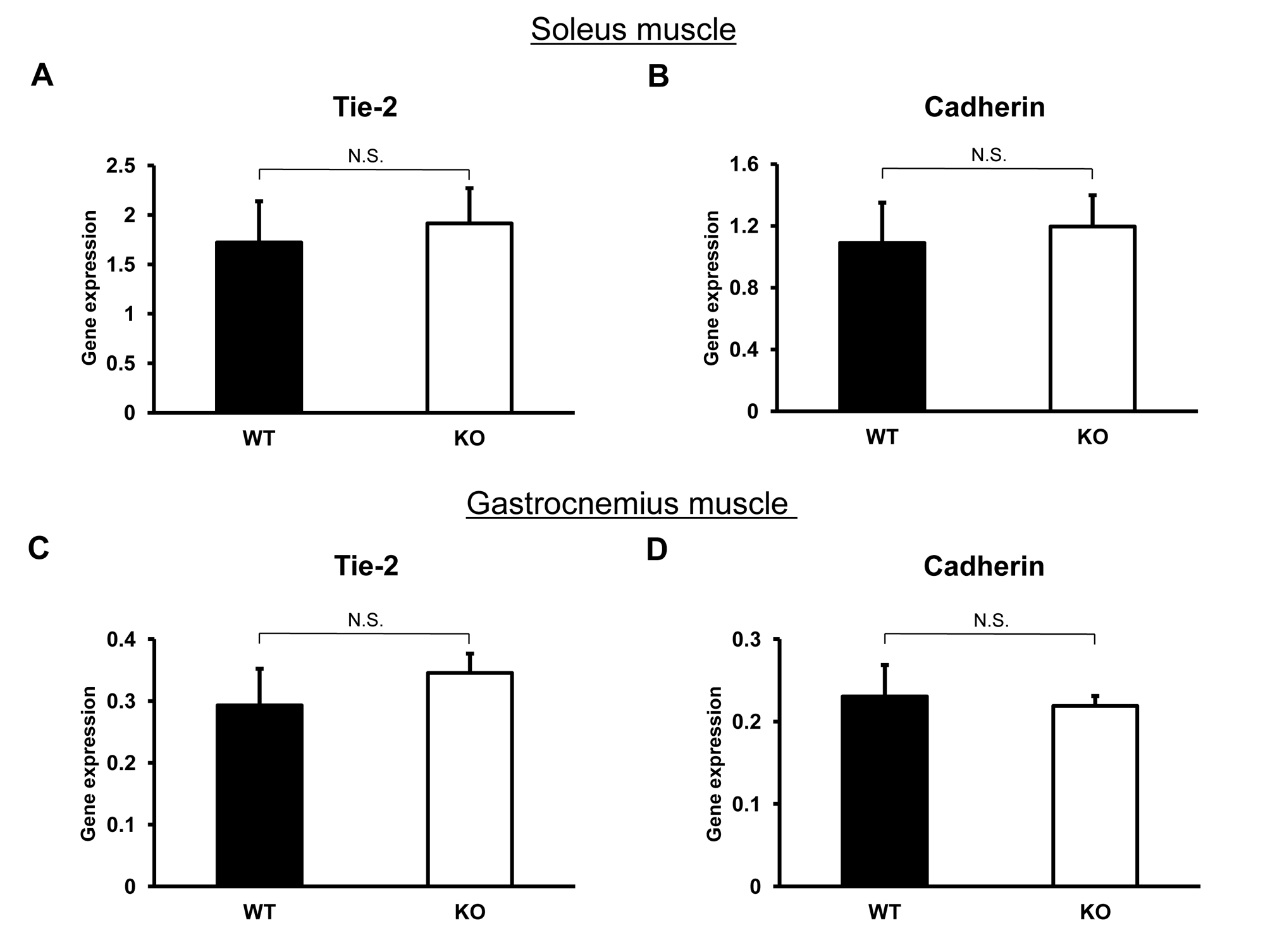

Supplement: Figure S3 — Endothelial gene expression in skeletal muscle of TIMP-3 KO and wild type mice. Expression of Tie-2 (A) and Cadherin (B) in soleus muscle, and Tie-2 (C) and Cadherin (D) in gastrocnemius muscle, in TIMP-3 knockout (KO) and wild type (WT) mice is presented as mean ± SD (n = 6–7/group). (TIF) [file pone.0094930.s003.tif]

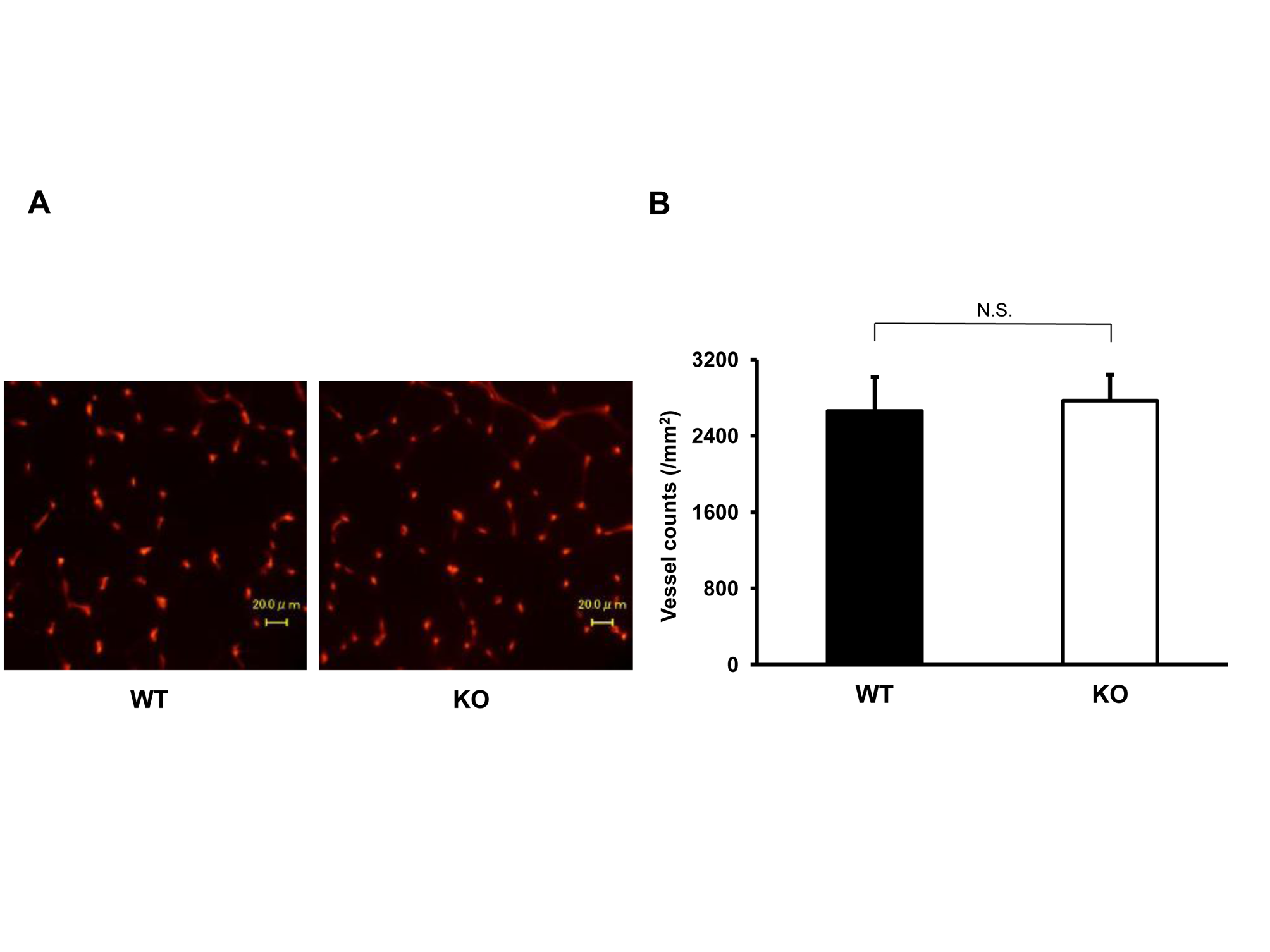

Supplement: Figure S4 — Vessel counts in gastrocnemius muscle of TIMP-3 KO and wild type mice. Immunostaining for CD31, an endothelial cell marker (A), and quantification of vessels based on CD31 expression (B) in soleus muscle of TIMP-3 knockout (KO) and wild type mice. (TIF) [file pone.0094930.s004.tif]

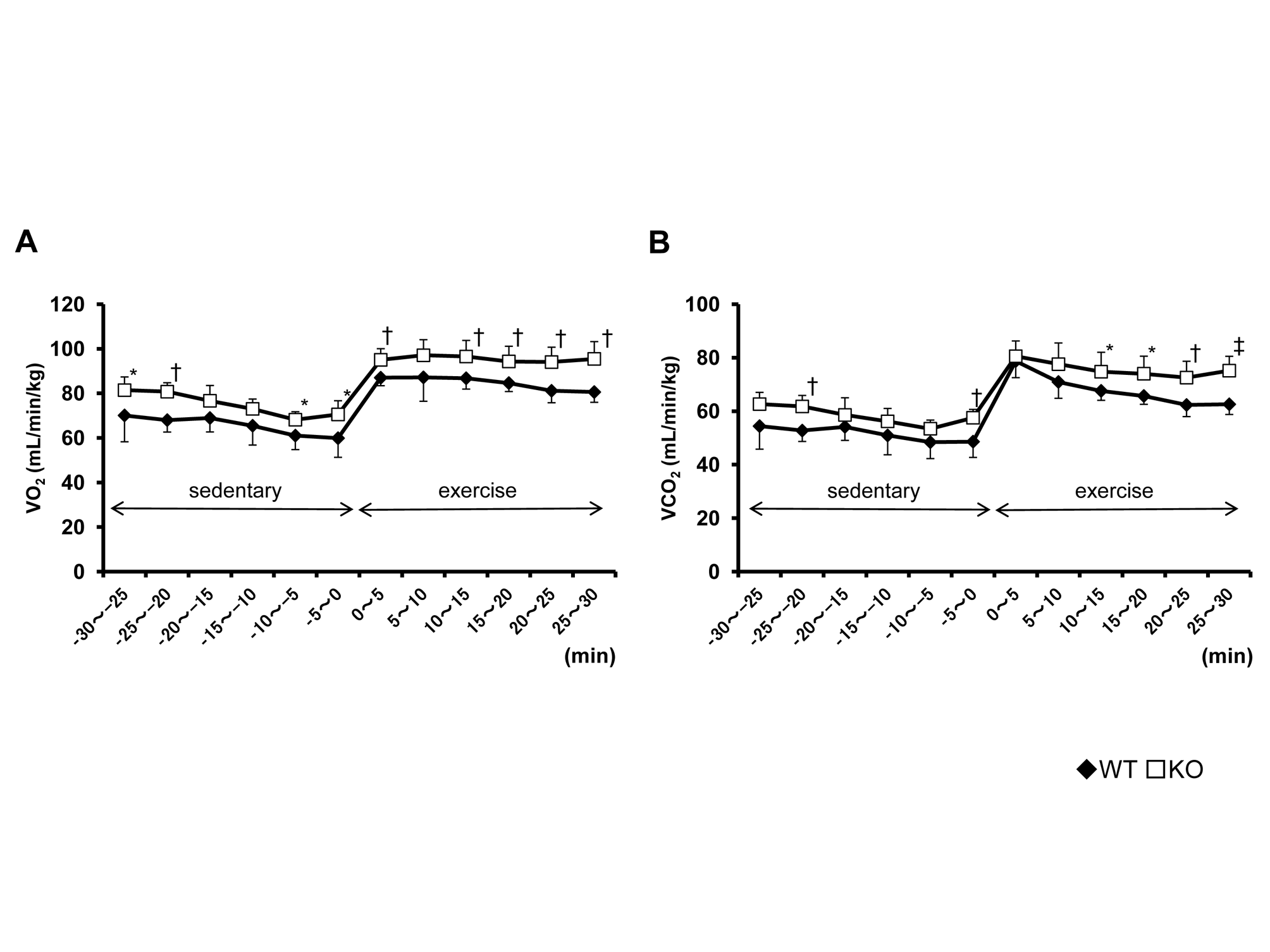

Supplement: Figure S5 — Respiratory parameters of TIMP-3 KO and wild type mice during the exercise performance test. Oxygen consumption (VO2) (A) and carbon dioxide production (VCO2) (B) in TIMP-3 knockout (KO) and wild type (WT) mice are presented as mean ± SD (n = 7/group). *p<0.05, †p<0.01, ‡p<0.001. (TIF) [file pone.0094930.s005.tif]

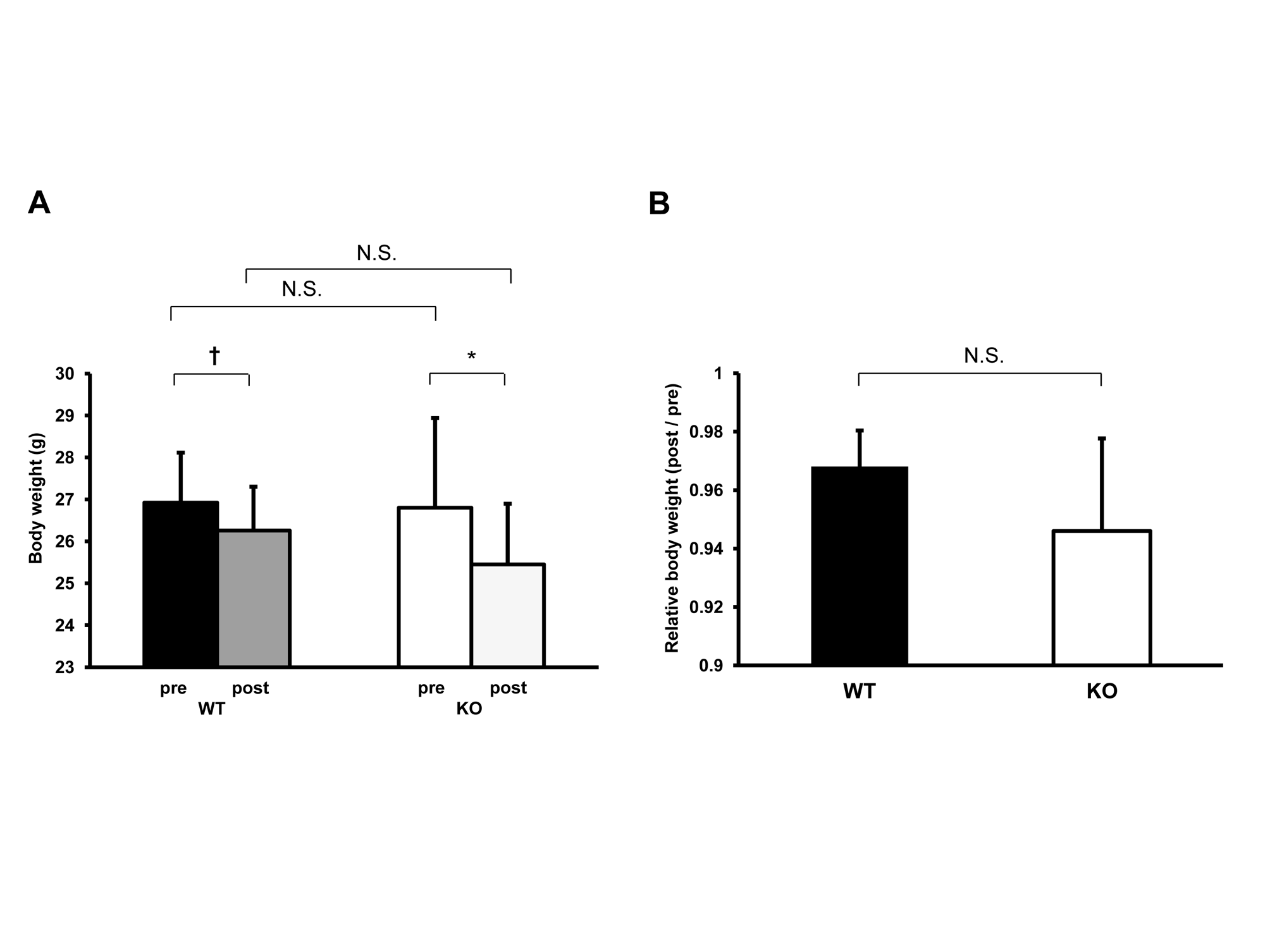

Supplement: Figure S6 — Body weight of TIMP-3 KO and wild type mice before and after exercise. Net body weight (mean ± SD) (A) and body weight relative to before exercise (mean ± SD) (B) are presented (n = 8/group). (TIF) [file pone.0094930.s006.tif]

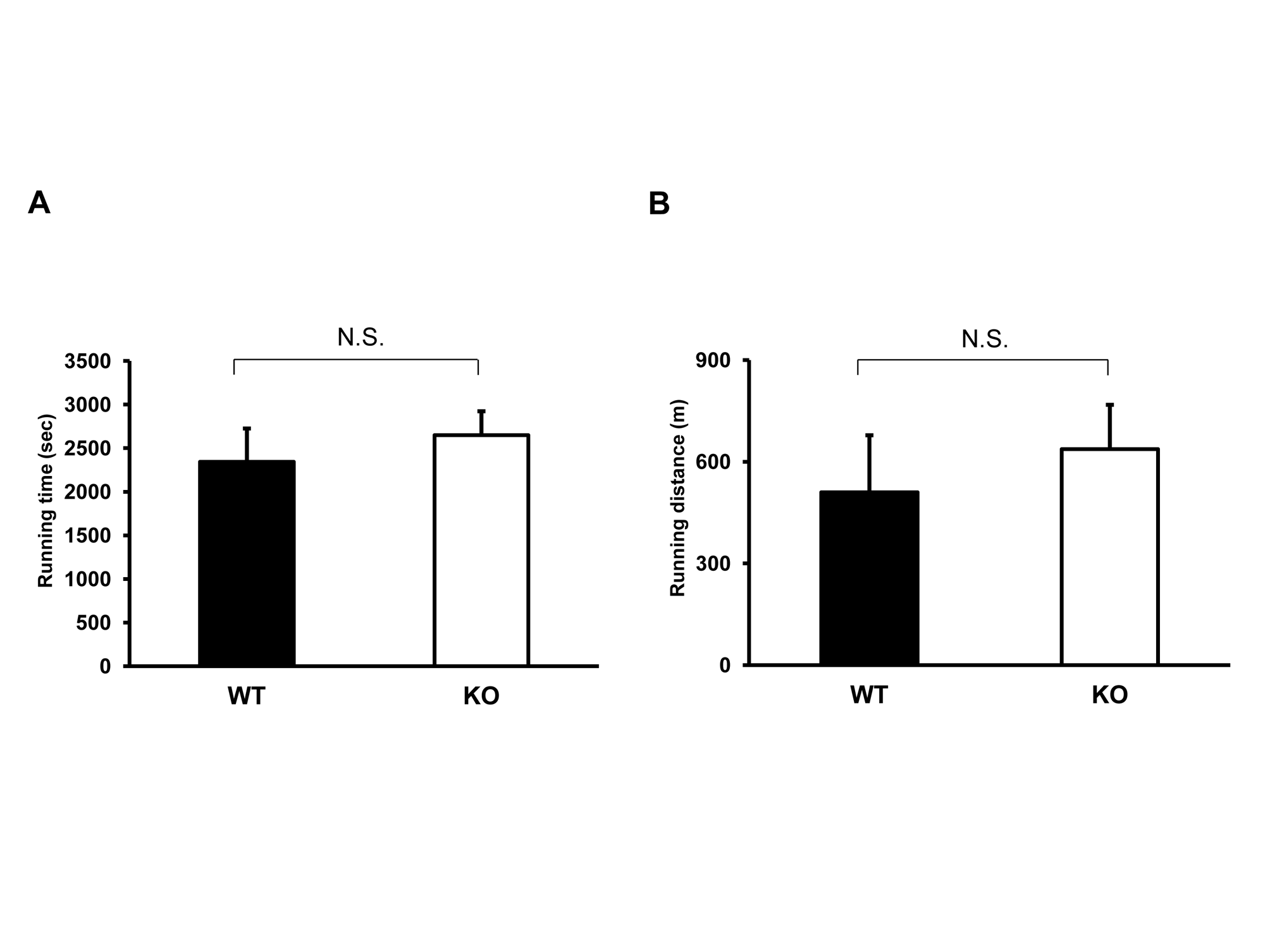

Supplement: Figure S7 — Physical endurance in TIMP-3 KO and wild type mice. Running time (A) and distance (B) for TIMP-3 knockout (KO) and wild type (WT) mice are presented as mean ± SD (n = 8/group). (TIF) [file pone.0094930.s007.tif]
