# Supplementary material for: Tissue Inhibitor of Metalloproteinase-3 Knockout Mice Exhibit Enhanced Energy Expenditure through Thermogenesis
Source: PLoS One. 2014 Apr 15;9(4):e94930. doi: 10.1371/journal.pone.0094930 (PMC3988092; doi:10.1371/journal.pone.0094930)
Supplement: Text S1 — Protocol of exercise performance test and Treadmill-based physical training. (DOCX) [file pone.0094930.s008.docx]

**Text S1. Supporting methods**

*Exercise performance test –* Eight-month-old mice were acclimated to an air-tight treadmill chamber (Model MK-680AT/02M, Muromachi Kikai, Tokyo, Japan), followed by measurements of VO_2_, VCO_2_ and RER in a sedentary state (air flow rate, 0.90 L/min) for 30 min. Mice then exercised on a treadmill at a speed of 10 m/min, and VO_2_, VCO_2_ and RER were measured for 30 min.

*Treadmill-based physical training –* Fifteen-week-old mice were trained 5 days/week on a treadmill for two weeks. On the first two days, animals were given a 20 min warm-up period with slow walking speeds for acclimation. Training began at a pace of 10 m/min, a moderate walk-jog pace, for 20 min. As mice became increasingly familiar with the treadmill, the velocity was increased to 15 m/min and then in 2 m/min increments at 5 min intervals until the velocity reached 25 m/min; 25–28 m/min roughly corresponds to 75–80% of maximal oxygen consumption for mice [[1](#_ENREF_1)]. Endurance was measured on the next day of the training period according to the above training protocol until mice were exhausted. Exhaustion was determined as the point at which mice fell onto the metal grid more than three times within 10 sec or remained on the metal grid for more than 15 sec.

**Reference**

1. Billat VL, Mouisel E, Roblot N, Melki J (2005) Inter- and intrastrain variation in mouse critical running speed. J Appl Physiol 98: 1258-1263.
